# Multiple Ways for Stress Sensing and Regulation of the Endoplasmic Reticulum-stress Sensors

**DOI:** 10.1247/csf.21015

**Published:** 2021-03-26

**Authors:** Quynh Giang Le, Yukio Kimata

**Affiliations:** 1 Institute of Biomedicine and Pharmacy, Vietnam Military Medical University, 222 Phung Hung, Ha Dong, Ha Noi, Vietnam; 2 Institute of Biotechnology, Vietnam Academy of Science and Technology, 18 Hoang Quoc Viet road, Cau Giay, Ha Noi, Vietnam; 3 Graduate School of Science and Technology, Nara Institute of Science and Technology, 8916-5 Takayama, Ikoma, Nara, 630-0192, Japan

**Keywords:** endoplasmic reticulum, stress, unfolded protein response, molecular chaperone

## Abstract

Dysfunction of the endoplasmic reticulum (ER), so-called ER stress, is accompanied with accumulation of unfolded proteins in the ER. Eukaryotic cells commonly have an ER-located transmembrane protein, Ire1, which triggers cellular protective events against ER stress. In animal cells, PERK and ATF6 also initiate the ER-stress response. As a common strategy to control the activity of these ER-stress sensors, an ER-resident molecular chaperone, BiP, serves as their negative regulator, and dissociates from them in response to ER stress. Although it sounds reasonable that unfolded proteins and Ire1 compete for BiP association, some publications argue against this competition model. Moreover, yeast Ire1 (and possibly also the mammalian major Ire1 paralogue IRE1α) directly detects ER-accumulated unfolded proteins, and subsequently oligomerizes for its further activation. Apart from protein misfolding, the saturation of membrane phospholipids is another outcome of ER-stressing stimuli, which is sensed by the transmembrane domain of Ire1. This review describes the canonical and up-to-date insights concerning stress-sensing and regulatory mechanisms of yeast Ire1 and metazoan ER-stress sensors.

## Introduction

The endoplasmic reticulum (ER) is a membrane-bound organelle in which most of the secretory and transmembrane proteins are folded and assembled in eukaryotic cells. Once properly matured in the ER, proteins are packed into transport vesicles and carried to the cell surface or other organelles via the Golgi apparatus. Dysfunction of the ER is thus associated with accumulation of unfolded proteins in the ER, and is now collectively called ER stress. Kozutsumi *et al.* (1988) reported that, in mammalian cells, ER stress results in transcriptional induction of some ER-located proteins including BiP, which is a molecular chaperone belonging to the heat shock protein 70 (HSP70) family (Behnke *et al.*, 2015). This cellular response is conserved throughout eukaryotic species, and is known as the ER stress response or the unfolded protein response (UPR).

**Fig. 1:**
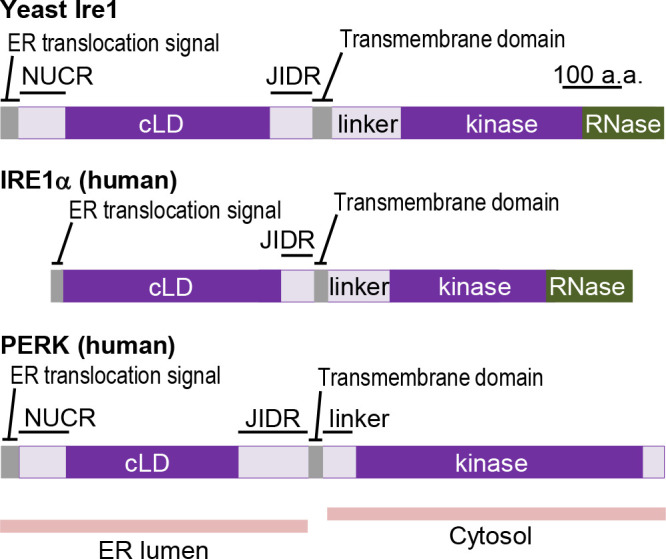
Structure of Ire1 and PERK. Unlike those of the NUCR or the JIDR, the amino-acid sequences of the cLD are weakly but significantly conserved among these proteins. In our previous publication (Kimata *et al.*, 2004), the NUCR, the cLD and the JIDR of the yeast Ire1 were named as Subregions I, II–IV and V.

**Fig. 2:**
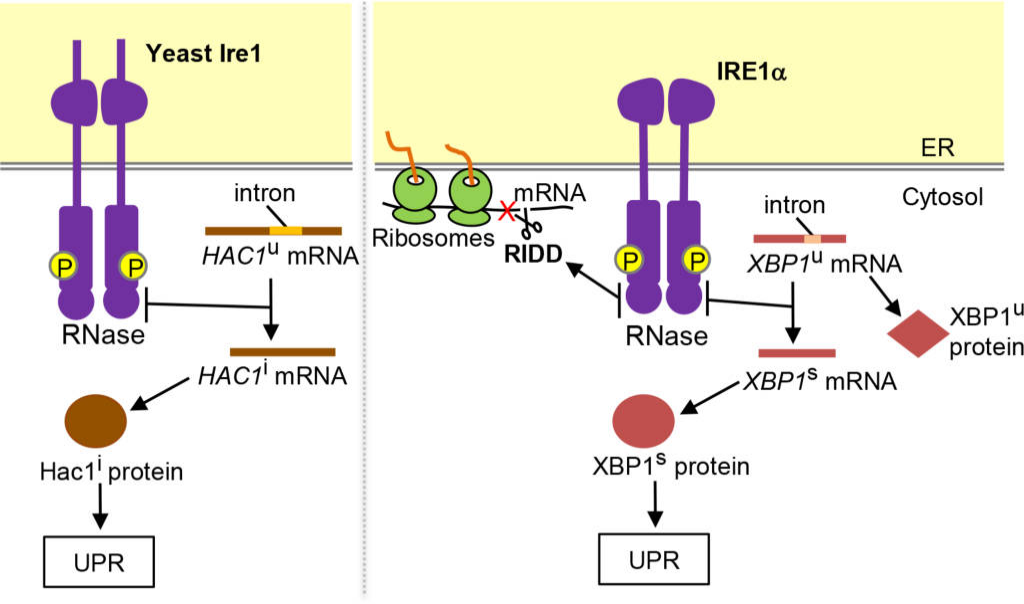
Functions of yeast Ire1 and mammalian IRE1α. Self-association and auto-phosphorylation of Ire1 lead to its activation as RNase, which evokes the indicated events. While, as described later in this article, Ire1 is more strongly activated as high-order oligomers, here it is presented as dimers. Although not shown in this figure, it is also likely that IRE1α phosphorylates other proteins.

The intracellular signaling pathway of the UPR has been initially uncovered through genetic studies using *Saccharomyces cerevisiae* (hereafter simply called yeast) as a model organism. As illustrated in Fig. 1, an ER-located type-I transmembrane protein Ire1 carries two enzymatic motifs as an endoribonuclease (RNase) and a Ser/Thr protein kinase, and triggers the UPR (Cox *et al.*, 1993; Mori *et al.*, 1993; Shamu and Walter, 1996; Sidrauski and Walter, 1997). At least in yeast cells, it appears unlikely that Ire1 phosphorylates another protein(s). Upon ER stress, yeast Ire1 is *trans*-autophosphorylated, which means that one Ire1 molecule phosphorylates another self-associated Ire1 molecule (Shamu and Walter, 1996). Yeast Ire1 is then activated as an RNase to promote *HAC1*-mRNA splicing for conversion of the precursor *HAC1*u form to the mature *HAC1*i form (Fig. 2; Cox and Walter, 1996; “u” and “i” respectively stand for “uninduced” and “induced”). The *HAC1*i mRNA is then translated into a transcription factor protein that induces various genes including that encoding BiP.

Ire1 is conserved throughout eukaryotic species. Metazoan Ire1 orthologues including the major vertebrate Ire1 paralogue, namely IRE1α (Fig. 1), promote the splicing of X-box binding protein 1 (XBP1) mRNA (XBP1u; “u” stands for “unspliced”), the mature form of which (XBP1s; “s” stands for “spliced”), as well as that of the yeast *HAC1* mRNA, is translated into a transcription factor (Fig. 2; Yoshida *et al.*, 2001; Calfon *et al.*, 2002). Meanwhile, unlike yeast Ire1, metazoan Ire1 orthologues degrade mRNAs encoding proteins that traverse the ER (Fig. 2; Hollien and Weissman, 2006; Maurel *et al.*, 2014). This phenomenon is known as the regulated Ire1-dependent decay (RIDD), and is thought to attenuate protein load into the ER upon ER stress. Vertebrate cells also carry a minor Ire1 paralogue named as IRE1β, which, in mouse body, is expressed only in mucin-secreting cells (Tsuru *et al.*, 2013). Ishikawa *et al.* (2017) reported that IRE1β performs *XBP1*-mRNA splicing, whereas other publications suggested that this is not the main role of IRE1β (Tsuru *et al.*, 2013; Grey *et al.*, 2020). It is also likely that these IRE1 paralogues activate c-Jun N-terminal kinases (JNKs) independently of their RNase activity (Urano *et al.*, 2000).

In vertebrate cells, another ER-located transmembrane protein, the activating transcription factor (ATF) 6, also serves as an ER-stress sensor. Upon ER stress, ATF6 is transported from the ER to the Golgi apparatus, in which ATF6 is cleaved and becomes a soluble and active transcription factor ATF6(N) (“N” stands for “N-terminal”; Haze *et al.*, 1999; Yoshida *et al.*, 2000; Ye *et al.*, 2000; Chen *et al.*, 2002). XBP1s and ATF6 transcriptionally induce partly overlapping genes, many of which encode proteins working for the ER (Shoulders *et al.*, 2013). In addition, the XBP1 gene is transcriptionally induced by ATF6 (Yoshida *et al.*, 2001).

Metazoan cells carry another ER-located transmembrane kinase, PKR-like endoplasmic reticulum kinase (PERK), the luminal-domain sequence of which exhibits a weak but significant homology with that of the aforementioned Ire1 orthologues and paralogues (Fig. 1; Harding *et al.*, 1999; Liu *et al.*, 2000). Under ER-stressing conditions, PERK phosphorylates and inactivates the α subunit of the eukaryotic initiation factor 2 (eIF2α). While this leads to a global decrement of protein synthesis, which can alleviate ER stress, it is also known that PERK evokes more sophisticated and complicated cellular events (Rozpedek *et al.*, 2016). The phosphorylation of eIF2α facilitates translation of ATF4 mRNA by skipping its upstream open reading frame, resulting in the initiation of a gene expression program named as the integrated stress response (Harding *et al.*, 2000; Donnelly *et al.*, 2013). The C/EBP homologous protein (CHOP), which contributes to ER stress-induced apoptosis, is one of the downstream induction targets of ATF4 (Zinszner *et al.*, 1998; Harding *et al.*, 2000).

How is ER stress sensed by Ire1 orthologues and PERK? Studies on yeast Ire1 initially provided various intriguing insights to answer this question. Meanwhile, recent biochemical approaches using recombinant IRE1α and PERK fragments have provided new views, which are sometimes controversial. This review discusses canonical and current understanding of the molecular mechanisms by which they sense ER stress and are regulated. Furthermore, the ER stress-sensing and regulation mechanisms of ATF6 are also described.

## Stress-sensing and regulatory mechanism of yeast Ire1

Since Ire1 is an ER-located transmembrane protein, it is widely believed that its luminal moiety is responsible for controlling Ire1’s activity in response to ER conditions. Several experimental data and a computer prediction cumulatively indicate that the luminal moiety of yeast Ire1 has two loosely folded segments (Kimata *et al.*, 2004; Oikawa *et al.*, 2005; Credle *et al.*, 2005; Mathuranyanon *et al.*, 2015), namely the N-terminal unconserved region (NUCR; Fig. 1) and the juxtamembrane intrinsically disordered region (JIDR; Fig. 1). Meanwhile, the central segment of the luminal domain, which is called the core stress-sensing region (CSSR) or the core luminal domain (cLD; Fig. 1), is tightly folded and responsible for the self-association of Ire1 (Credle *et al.*, 2005; Kimata *et al.*, 2007).

Okamura *et al.* (2000) showed that BiP is associated with yeast Ire1 under normal conditions and dissociates from it in response to ER stress. Moreover, yeast cells only weakly induced the UPR even under ER-stress conditions when carrying BiP mutations that stabilize the BiP-Ire1 association (Kimata *et al.*, 2003). These observations suggest a role of BiP as a negative regulator of Ire1. Based on deletion mutation analyses, we and others have proposed that the BiP-binding site is located on the JIDR of yeast Ire1 (Kimata *et al.*, 2004; Pincus *et al.*, 2010). Supposing that Ire1 is captured by BiP as a chaperone substrate, it seems reasonable that the BiP-binding site is intrinsically disordered. Albeit less tightly than the wild type, yeast Ire1 mutants not carrying the JIDR are regulated depending on ER stress (Kimata *et al.*, 2004; Pincus *et al.*, 2010). Therefore, it is unlikely that BiP is the sole master regulator of yeast Ire1.

**Fig. 3:**
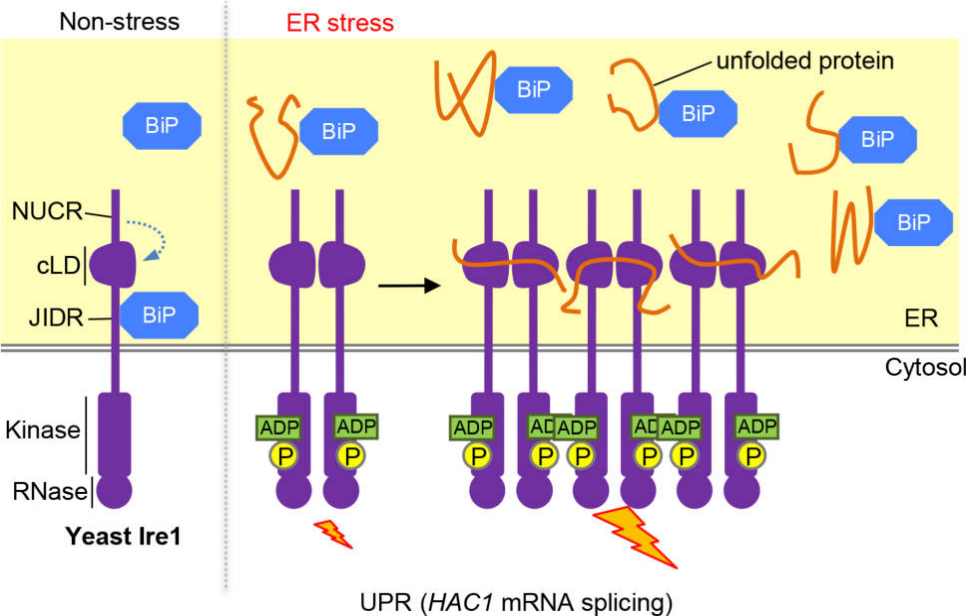
Our model for upregulation of Ire1 upon ER accumulation of unfolded proteins in yeast cells. Under non-stress conditions, the JIDR (the BiP-binding site) and the NUCR inhibit the self-association of Ire1. Upon ER stress, BiP is dissociated from Ire1, which is then dimerized. The Ire1 dimers are automatically phosphorylated and then captures ADP for their activation as RNase. Meanwhile, the Ire1 dimers directly captures ER-accumulated unfolded proteins and are further bundled, leading to more potent UPR.

The NUCR also contributes to suppression of the activity of yeast Ire1 in non-stressed cells (Oikawa *et al.*, 2007; Mathuranyanon *et al.*, 2015). Nevertheless, it is unlikely that BiP binds to the NUCR (Kimata *et al.*, 2004; Oikawa *et al.*, 2007). According to Mathuranyanon *et al.* (2015), the NUCR intramolecularly interacts with the cLD, inhibiting the cLD-dependent self-association of Ire1 under normal conditions. A yeast Ire1 mutant carrying neither the JIDR nor the NUCR, which here we call ΔNUCR/ΔJIDR Ire1, was self-associated probably as a dimer even under normal conditions (Oikawa *et al.*, 2007; Mathuranyanon *et al.*, 2015). This observation suggested that the BiP dissociation from Ire1 contributes to, although not sufficient for, the dimerization of Ire1 under ER-stress conditions (Fig. 3). Since ΔNUCR/ΔJIDR Ire1 was constitutively auto-phosphorylated in yeast cells, we deduce that Ire1 is automatically auto-phosphorylated when dimerized (Fig. 3; Le *et al.*, 2021).

When yeast cells are strongly ER-stressed, Ire1 exhibits a punctate-like distribution (Kimata *et al.*, 2007; Aragón *et al.*, 2009; Ishiwata-Kimata, 2013b). Since this ER stress-dependent localization change, namely the cluster formation of Ire1, was observable even when Ire1 carries the ΔNUCR/ΔJIDR mutation (Mathuranyanon *et al.*, 2015), we think that neither the NUCR nor the JIDR is involved in the regulation of this process. Meanwhile, the Ire1 cluster formation is likely based on the properties of the cLD. According to X-ray crystallographic analysis (Credle *et al.*, 2005), the cLD dimer of yeast Ire1 forms a groove-like structure that resembles that of the peptide-binding groove of the major histocompatibility complex I. Further studies by us and others indicated that the cLD actually captures unfolded proteins, leading to its high-order oligomerization (Fig. 3; Kimata *et al.*, 2007; Gardner and Walter, 2011; Promlek *et al.*, 2011). According to Korennykh *et al.* (2009), clustered Ire1 molecules exert a stronger RNase activity than Ire1 dimers. Moreover, *HAC1* mRNA is actively gathered at the Ire1 foci in yeast cells (Aragón *et al.*, 2009; van Anken *et al.*, 2014). It is therefore likely that the Ire1 cluster formation leads to the efficient splicing of *HAC1* mRNA.

The multistep regulatory events on the ER-luminal side described so far are documented in more detail in our previous review articles (Kimata and Kohno 2011; Ishiwata-Kimata *et al.*, 2018), and are likely to contribute to the tight regulation of the UPR. Probably because a number of genes are directly or indirectly controlled by Ire1 and *HAC1* (Travers *et al.*, 2000; Kimata *et al.*, 2006), yeast cells expressing less tightly regulated mutant versions of Ire1 grew slowly (Mathuranyanon *et al.*, 2015; Le *et al.* 2021). Therefore, it should be important that Ire1 activity is quickly attenuated under the post-ER-stress phase of yeast cells. Ire1 mutants not carrying the JIDR were shown to be poorly downregulated when yeast cells were recovering from ER-stress conditions after a transient UPR evocation (Pincus *et al.*, 2010; Ishiwata-Kimata *et al.*, 2013a). This observation strongly suggests that the Ire1 re-association with BiP largely contributes to the attenuation of its activity.

As aforementioned, auto-phosphorylation of yeast Ire1 leads to its activation as an RNase (Shamu and Walter, 1996; Lee *et al.*, 2008). While, to promote a phospho-transfer reaction, a kinase carries a nucleotide-binding pocket (NBP) to which ATP binds, the NBP of Ire1 has another function. The NBP of auto-phosphorylated Ire1 captures ADP, leading to a further conformational change in the cytosolic moiety of Ire1, which then exhibits higher activity as an RNase (Lee *et al.*, 2008; Korennykh *et al.*, 2009; Korennykh *et al.*, 2011). In other words, ADP serves as an activation ligand of Ire1. Intriguingly, kinase-dead mutants of Ire1 are thus able to splice *HAC1* mRNA and to induce the UPR in yeast cells when undergone a similar conformational change (Papa *et al.*, 2003; Chawla *et al.*, 2011; Rubio *et al.*, 2011). According to our recent observation, an Ire1 mutant that is activated without undergoing these kinase domain-dependent regulatory events was hypersensitive to ER stress, suggesting a role of the kinase domain for fine tuning of the UPR level (Le *et al.*, 2021).We assume that under mild ER-stress conditions, Ire1 is activated only weakly, partly because cells are healthy enough to perform ATP biogenesis normally, keeping a low cytosolic ADP level. In contrast, strong ER-stressing stimuli severely damage cells, elevating cytosolic ADP level and eventually activating Ire1 strongly (Le *et al.*, 2021).

Membrane-lipid biogenesis is another important role of the ER. Cox *et al.* (1997) exhibited an UPR induction by depletion of inositol, which is an important constituent of membrane lipid, from yeast culture. To our knowledge, this was the first report to argue that a membrane lipid-related abnormality causes ER stress and activates Ire1. Moreover, saturation of membrane lipids is now known to be an ER-stressing stimulus in yeast and mammalian cells (Pineau and Ferreira, 2010). Ire1 is also activated by imbalanced composition of hydrophilic moieties of phospholipids (low phosphatidyl choline (PC) and high phosphatidyl ethanolamine) in yeast cells (Thibault *et al.*, 2012). Micoogullari *et al.* (2020) reported a UPR induction upon defects of the very-long-chain fatty acid (VLCFA) metabolism. We think that induction of the UPR when yeast cells are confronted with these membrane lipid-related abnormalities is a reasonable cellular response, because the Ire1-*HAC1* pathway transcriptionally induces a large number of genes including those encoding enzymes that work for metabolism of membrane lipids or inositol (Travers *et al.*, 2000; Kimata *et al.*, 2006).

**Fig. 4:**
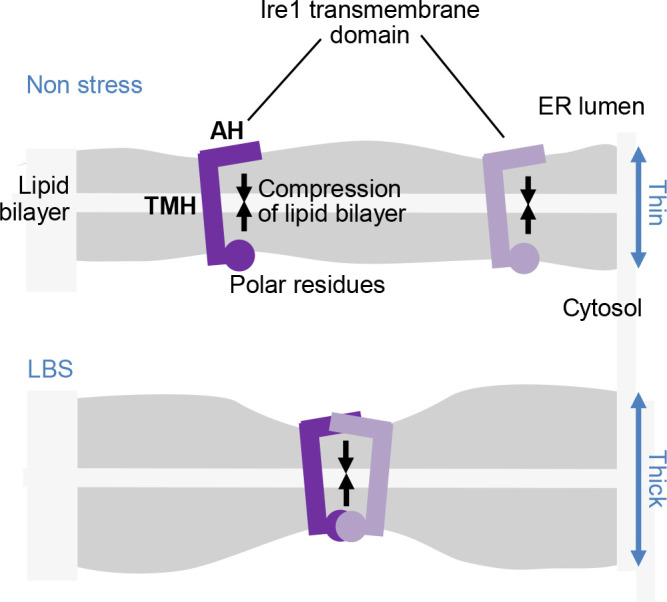
Possible involvement of the Ire1 transmembrane-domain structure in the activation of Ire1 upon LBS. The short TMH of Ire1 is juxtaposed N-terminally to the AH and C-terminally to polar residues, causing compression of the lipid bilayer (Halbleib *et al.*, 2017; Covino *et al.*, 2018). When the lipid-bilayer structure is disturbed, for example, through membrane-lipid saturation, free energy cost for the lipid-bilayer compression by the Ire1 transmembrane domain is increased. Ire1 molecules are then gathered to cope with this situation.

In yeast cells, defects in the PC biogenesis or the VLCFA metabolism lead to saturation of the fatty-acid moieties of phospholipids (Boumann *et al.*, 2006; Micoogullari *et al.*, 2020). Therefore, it is possible that membrane lipid-related abnormalities, which is now collectively called the lipid bilayer stress (LBS), activate Ire1 commonly via membrane-lipid saturation. Mutation analyses of yeast Ire1 demonstrated that LBS and ER accumulation of unfolded proteins are different types of ER-stressing stimuli that activate Ire1 in different ways (Promlek *et al.*, 2011; Halbleib *et al.*, 2017; Tran *et al.*, 2019, Ho *et al.*, 2020). According to Promlek *et al.* (2011) and Ho *et al.* (2020), the ER-luminal moiety of Ire1 is dispensable for UPR induction in response to LBS. An *in vitro* experiment described by Volmer *et al.* (2013) showed that the auto-phosphorylation degree of recombinant fragments of IRE1α or PERK was enhanced when they were incorporated into liposomes highly containing saturated phospholipids. It is thus likely that Ire1 orthologues and PERK directly sense the saturation degree of membrane lipids. The short transmembrane helix (TMH) of yeast Ire1 is juxtaposed to an amphipathic helix (AH), which contributes to LBS sensing (Fig. 4; Halbleib *et al.*, 2017; Tran *et al.*, 2019; Ho *et al.*, 2020). According to Halbleib *et al.* (2017) and Covino *et al.* (2018), because of this unique structure, Ire1 molecules compress the lipid bilayer, and gather upon LBS (Fig. 4). Unlike the case of robust accumulation of unfolded proteins in the ER, Ire1 does not seem to cluster upon LBS (Ho *et al.*, 2020).

However, it may also be possible that disturbance of the membrane-lipid homeostasis worsens the protein-folding status in the ER, leading to the UPR evocation. Shyu Jr *et al.* (2019) reported that, in yeast cells, LBS causes destabilization and degradation of ER-resident transmembrane proteins, which may further damage the ER.

One question emerging from these insights is what (LBS or ER accumulation of unfolded proteins) mediates Ire1 activation by a stressing stimulus. This question can be answered using Ire1 mutants that are impaired in one of the aforementioned stress-sensing mechanisms. The thiol-reducing reagent dithiothreitol (DTT) has been believed to activate Ire1 by damaging the protein-folding status of ER-client proteins. However, it is also likely that LBS is partially involved in the DTT-induced UPR evocation in yeast cells (Halbleib *et al.*, 2017; Tran *et al.*, 2019). According to Reinhard *et al.* (2020), the unsaturation level of membrane lipids is decreased when yeast cells are exposed to DTT. Another intriguing example is ethanol, which induces ER stress in animal and yeast cells (Miyagawa *et al.*, 2014; Yang and Luo, 2015). It is likely that ethanol causes both protein denaturation and membrane fluidification (Miyagawa *et al.*, 2014; Navarro-Tapia *et al.*, 2018). According to mutation analysis of yeast Ire1 (Tran *et al.*, 2019), ethanol activates Ire1 via both LBS and ER accumulation of unfolded proteins.

## Stress-sensing and regulatory mechanism of IRE1α and PERK

As well as yeast Ire1, the vertebrate Ire1 paralogues, IRE1α and IRE1β, and PERK are ER-located type-I transmembrane proteins. On the luminal side, they commonly carry weakly but significantly homologous cLD sequences (Fig. 1). Therefore, one may assume that they are controlled similarly to yeast Ire1. Hereafter, this review discusses if this assumption is true or not.

Bertolotti *et al.* (2000) demonstrated that in mammalian cells, BiP is associated with IRE1α (and also IRE1β), whereas the heteromeric complexes are dissociated in response to ER stress. Similarly to yeast Ire1, IRE1α carries the JIDR, which is dispensable for its activity (Fig. 1; Oikawa *et al.*, 2009). Albeit partly, a deletion mutation of the JDIR compromised the BiP association with IRE1α and activated IRE1α (Oikawa *et al.*, 2009). This observation suggests a role of BiP as a negative regulator of IRE1α. Meanwhile, it is likely that BiP is also targeted on the cLD (Carrara *et al.*, 2015b; Amin-Wetzel *et al.*, 2019).

**Fig. 5:**
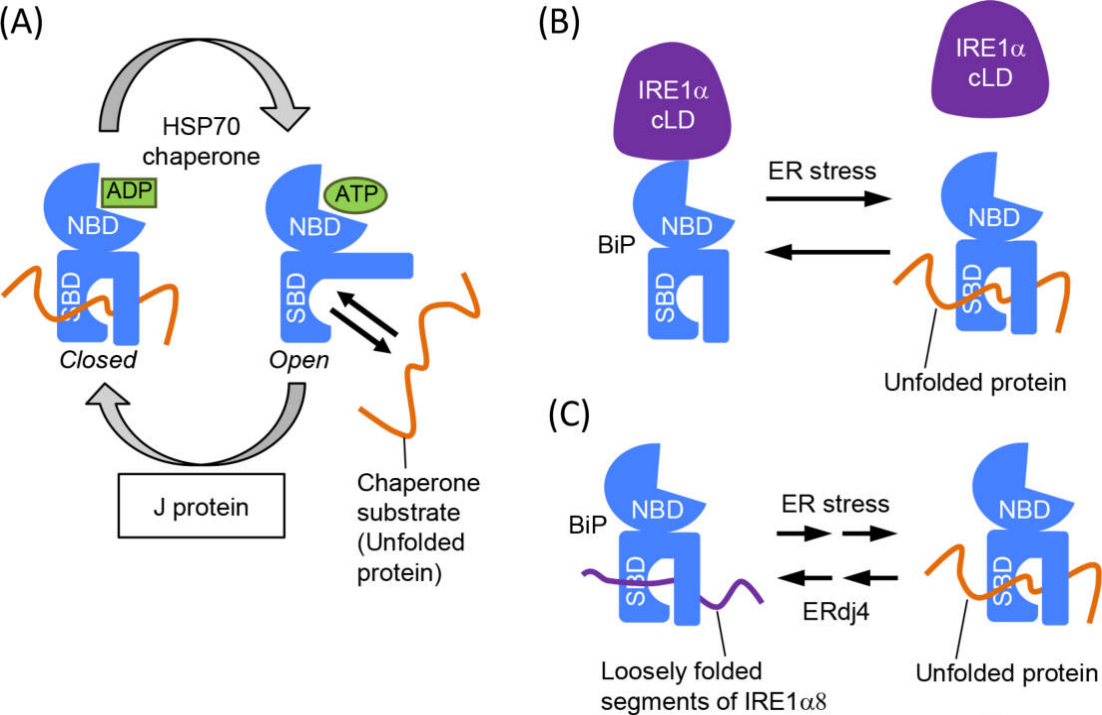
Relationship between BiP and Ire1. (A) In general, the HSP70-family molecular chaperones capture client substrate proteins dependently on its nucleotide-binding states. (B) and (C) As described in the main text, two theories are proposed to explain the association/dissociation mode between IRE1α and ER-accumulated unfolded proteins. Nucleotides bound to BiP are not shown in panels B and C.

As reviewed in Behnke *et al.* (2015), BiP is the ER-located version of the HSP70-family molecular chaperone, which is composed of the nucleotide-binding domain (NBD) and the substrate-binding domain (SBD). In general, association between the HSP70-family chaperone and unfolded-protein substrates is regulated by the ATPase cycle (Fig. 5A). When ATP is bound to the NBD, the SBD is the “open” form, which captures and releases substrate peptides rapidly. J proteins promote hydrolysis of ATP bound to the NBD, and turn the SBD into the “closed” form, which tightly captures substrate proteins. It is also widely accepted that a J protein facilitates the interaction between a HSP70-family chaperone and a specific chaperone substrate(s). Through the nucleotide exchange on the NBD (from ADP to ATP), the SBD returns to the “open” form.

Recently, the interaction between BiP and IRE1α has been more deeply investigated, which has led to two controversial theories. Carrara *et al.* (2015b) reconstituted the BiP-cLD heteromeric complex, the formation of which was abolished in the presence of a model unfolded peptide, in their *in vitro* experiments. According to the observations from this and the following studies (Carrara *et al.*, 2015b; Kopp *et al.*, 2018; Kopp *et al.*, 2019), IRE1α is not a chaperone substrate of BiP, but binds to the NBD of BiP. As illustrated in Fig. 5B, this allosteric regulation model argues that the BiP NBD is dissociated from IRE1α when its SBD captures unfolded-protein substrates. Todd-Corlett *et al.* (2007) suggested that yeast Ire1 also binds to the NBD of BiP.

In contrast, the observations presented in Amin-Wetzel *et al.* (2019) strongly suggest that IRE1α is a chaperone substrate of BiP. The cLD carries a loosely folded segment, to which BiP binds (Amin-Wetzel *et al.*, 2019). According to this theory, IRE1α is dissociated from BiP upon ER stress through competition against unfolded proteins for BiP binding (Fig. 5C). While in mammalian cells, the ER contains various J proteins, among them, ERdj4 specifically acts to facilitate the interaction between IRE1α and BiP (Amin-Wetzel *et al.*, 2017; Amin-Wetzel *et al.*, 2019).

In both the allosteric and competition models, BiP, but not IRE1α *per se*, is the direct sensor for unfolded proteins that is responsible for activation of IRE1α upon ER stress. Alongside BiP dissociation, IRE1α is self-associated (probably dimerized) to evoke the downstream events. Zhou *et al.* (2006) and Oikawa *et al.* (2009) argued against the direct physical interaction between unfolded proteins and the cLD of IRE1α. According to the X-ray crystal structure analysis presented by Zhou *et al.* (2006), the groove-like structure of the IRE1α luminal domain is too narrow to directly capture unfolded proteins. Amin-Wetzel *et al.* (2017) reported that IRE1α is considerably activated in non-stressed mammalian cells when its association with BiP is impaired. In this context, BiP may be the principal regulator of IRE1α.

Meanwhile, Li *et al.* (2010) and Belyy *et al.* (2020) noted the ER stress-dependent cluster formation of IRE1α, together with its dissociation under the post-ER-stress phase, in mammalian cells. How is this process promoted and regulated? In contradiction with Zhou *et al.* (2006) and Oikawa *et al.* (2009), Karagöz *et al.* (2017) presented NMR data indicating a conformational shift of the IRE1α cLD dimers by a model unfolded peptide. Moreover, Sundaram *et al.* (2018) reported that IRE1α physically interacts with an ER-accumulated model unfolded protein in mammalian cells. Similarly to the cLD of yeast Ire1, the IRE1α cLD dimers may be bundled through their direct interaction with unfolded proteins (Karagöz *et al.*, 2017). Meanwhile, a model unfolded peptide was shown not to directly stimulate the dimerization of the luminal domain of IRE1α (Amin-Wetzel *et al.*, 2019). It is thus likely that free IRE1α cLD molecules are spontaneously dimerized, whereas oligomerization of the IRE1α cLD dimers is promoted by unfolded proteins.

**Fig. 6:**
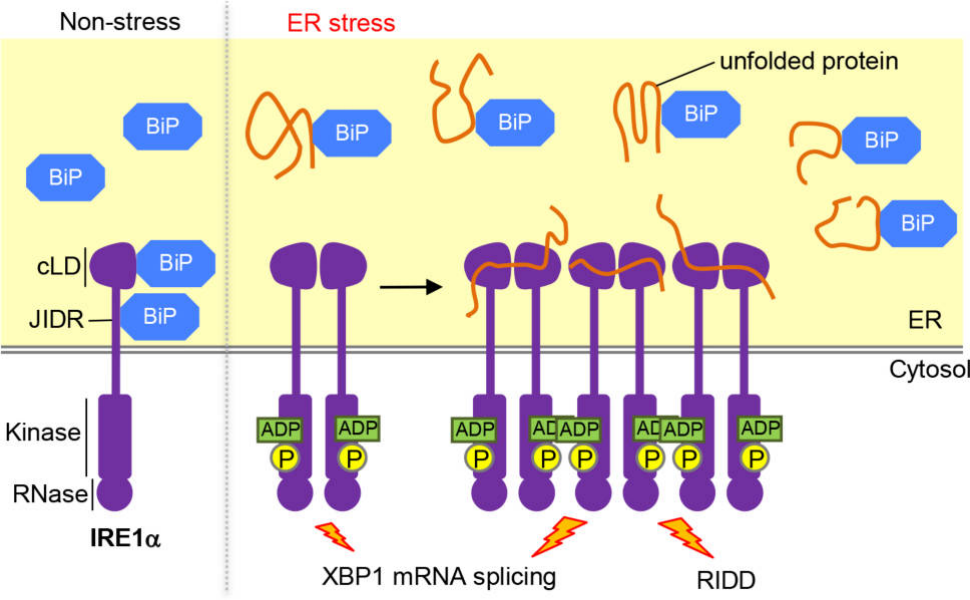
A possible model to explain how IRE1α is activated upon ER accumulation of unfolded proteins in animal cells. Under non-stress conditions, BiP is bound to the cLD and JIDR of IRE1α, which then stays non-self-associated. Upon ER accumulation of unfolded proteins, IRE1α dissociates from BiP and forms homo-dimers. It is uncertain if IRE1α and unfolded proteins bind to the same site of BiP. Similarly to those of yeast Ire1, IRE1αdimers are further bundled through their direct interaction with unfolded proteins. As described in the main text, it is possible that IRE1α dimers and oligomers have distinct biological functions.

Therefore, it is assumed that, while not carrying the NUCR (Fig. 1), IRE1α senses ER stress and is regulated in a fashion similar to that of yeast Ire1 as follows (Fig. 6). Under normal conditions, IRE1α is associated with BiP and remains non-self-associated. In response to ER stress, ER-accumulated unfolded proteins cause the dissociation of BiP from IRE1α, which then self-dimerizes (Bertolotti *et al.*, 2000). The dimerized IRE1α molecules directly capture unfolded proteins and are further oligomerized. Meanwhile, the cluster formation of IRE1α cannot be explained solely by the cLD oligomerization. According to Ricci *et al.* (2019), IRE1α clusters dependently on the auto-phosphorylation of its cytosolic moiety.

It should also be noted that IRE1α undergoes regulatory processes that have not been reported in the case of yeast Ire1. Eletto *et al.* (2014) proposed that IRE1α stably oligomerizes through its intramolecular disulfide-bond formation, which is cleaved by protein disulfide isomerase A6. Moreover, as reviewed by Hetz and Papa (2018), various proteins other than BiP have been reported to physically interact with and control IRE1α. One example is HSP47, which is known as the ER-located and collagen-specific molecular chaperone (Ito and Nagata, 2019). According to Sepulveda *et al.* (2018), the physical interaction between HSP47 and IRE1α promotes the dissociation of BiP from IRE1α and facilitates the self-association of IRE1α. In other words, HSP47 can serve as a positive regulator of IRE1α.

BiP is also associated with PERK. ER stress causes BiP dissociation from PERK, which seems to be linked to the self-oligomerization of PERK (Bertolotti *et al.*, 2000). As well as that of yeast Ire1, the luminal domain of PERK carries the NUCR and the JIDR, which function to downregulate PERK (Fig. 1; Ma *et al.*, 2002; Mathuranyanon *et al.*, 2015). In mammalian cells, the BiP association with PERK is almost completely abolished by a JIDR-deletion mutation of PERK (Ma *et al.*, 2002). Therefore, it is likely that BiP binds to the JIDR of PERK, which then remains non-self-associated, in unstressed cells. Meanwhile, an *in vitro* study performed by Carrara *et al.* (2015b) showed a physical interaction between BiP and the PERK cLD, which is inhibited by unfolded proteins. X-ray crystal structure analyses indicated PERK cLD’s tetrameric self-association, which contributes to the activation of PERK (Carrara *et al.*, 2015a; Wang *et al.*, 2016). Moreover, the PERK cLD has the ability to associate with unfolded proteins (Wang *et al.*, 2016), the biological meaning of which remains obscure. Taken together, although its binding site is not yet determined, BiP serves as a negative regulator of PERK and dissociates from PERK in response to ER stress. It is uncertain if the direct interaction between the PERK cLD and unfolded proteins contributes to the oligomerization and activation of PERK.

Even with mutations for deletion of their luminal domains, IRE1α and PERK responded to LBS that causes membrane-lipid saturation (Volmer *et al.*, 2013). This observation is consistent with the aforementioned insights from yeast Ire1 (Promlek *et al.*, 2011; Ho *et al.*, 2020). Halbleib *et al.* (2017) speculated that the scenario shown in Fig. 4 is also applicable to IRE1α and PERK, which, similarly to the yeast Ire1, carry AH sequences that are juxtaposed to TMH sequences. However, according to Kono *et al.* (2017), the AH sequence is dispensable for the activation of IRE1α by membrane-lipid saturation. It is therefore still obscure how LBS is sensed by the metazoan ER-stress sensors.

## Stress-sensing and regulatory mechanism of ATF6

Vertebrate species carry two ATF6 paralogues, namely ATF6α and ATF6β. Since the double knockout mutation of ATF6α and ATF6β, but not single knockout of either of the paralogues, of mouse causes embryonic lethality (Yamamoto *et al.*, 2007), they are, at least partly, functionally redundant. Meanwhile, the gene knockout study also indicated that ATF6α, but not ATF6β, is responsible for the transcriptional induction of ER quality control proteins (Yamamoto *et al.*, 2007). According to Thuerauf *et al.* (2004), the cytosolic segment of ATF6α acts as a transcription activator more strongly than that of ATF6β. It thus sounds rational that ATF6α were mainly employed to obtain the undermentioned insights concerning the activation mechanism of ATF6.

**Fig. 7:**
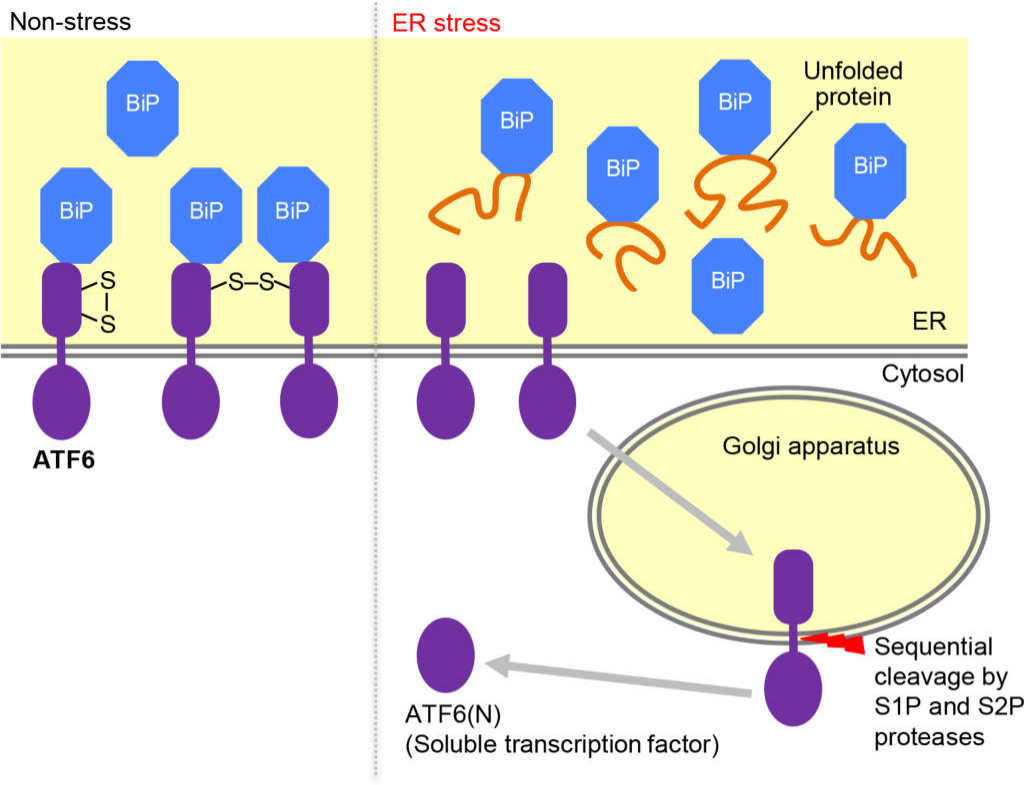
Activation process of ATF6. In non-stressed cells, ATF6 is retained in the ER as BiP-bound and oxidized forms. Upon ER stress, ATF6 dissociates from BiP and is reduced (cleavage of the disulfide bonds). The monomeric ATF6 proteins are then transported to the Golgi apparatus and proteolytically cleaved.

ATF6, a type-II transmembrane protein (Fig. 7), exhibits no sequence similarity to Ire1 or PERK, which are type-I transmembrane proteins. Therefore, it seems reasonable to assume that the regulation mechanism of ATF6 is completely different from that of Ire1 and PERK. However, as will be described later, it is also likely that, similarly to Ire1 and PERK, ATF6 is negatively regulated by BiP.

As aforementioned, ATF6 is retained in the ER of unstressed cells, and is transported to the Golgi apparatus, which carries the S1P and S2P proteases, in response to ER stress (Fig. 7; Ye *et al.*, 2000; Chen *et al.*, 2002; Shen *et al.*, 2002). ATF6 is then cleaved by these proteases to yield the soluble transcription activator ATF6(N) (Fig. 7; Haze *et al.*, 1999; Ye *et al.*, 2000). The regulated ER-to-Golgi transport is thus the key step for the ER stress-dependent activation of ATF6. According to Sato *et al.* (2011), the C-terminal luminal fragment of ATF6 (ATF6(C); “C” stands for “C-terminal”) underwent the regulated ER-to-Golgi transport in response to ER stress, even when it was produced in the ER of mammalian cells as a soluble protein. This observation indicates that the C-terminal luminal moiety of ATF6 alone is sufficient for sensing ER stress. The coat protein complex II (COPII) functions to bring out proteins from the ER for their transportation to the Golgi apparatus (Venditti *et al.*, 2014). An *in vitro* research performed by Schindler and Schekman (2009) reconstituted the COPII-dependent incorporation of ATF6 and ATF6(C) into membrane vesicles, which was stimulated by DTT.

Shen *et al.* (2002) indicated that ATF6 forms a hetero-complex with BiP, which is dissociated in response to ER stress. An ATF6 mutant that cannot bind to BiP was constitutively transported to the Golgi apparatus and cleaved (Shen *et al.*, 2002). Although this observation strongly suggests a role of BiP as a negative regulator of ATF6, it should also be noted that ATF6 luminal-domain mutations can affect the disulfide bond-formation status of ATF6, which, as described later, controls the ER-to-Golgi transport of ATF6. According to Shen *et al.* (2005), the ATF6-BiP complex is stable and does not carry out the association/dissociation cycle shown in Fig. 5A. This insight implies that the diminishment of the ATF6-BiP complex upon ER stress is not a result from simple competition between ATF6 and ER-accumulated unfolded proteins for binding to BiP. It thus remains unclear how ER stress leads to the dissociation of ATF6 from BiP.

Another topic is the disulfide-bond formation of ATF6. Schindler and Schekman (2009) showed that DTT leads ATF6 to dissociate from BiP and to exit from the ER in permeabilized cells. It is therefore possible that the protein redox state in the ER may directly affect the activation steps of ATF6. Compared to IRE1α or PERK, ATF6 seemed to respond to DTT-induced stress more efficiently than to other types of ER-stressing stimuli (DuRose *et al.*, 2006; Nadanaka *et al.*, 2007). According to Nadanaka *et al.* (2007) and Koba *et al.* (2020), ATF6 molecules are intermolecularly bridged by disulfide bonds to form self-dimers, or contain intramolecular disulfide bonds (Fig. 7). ER stress leads to reduction of the disulfide bonds of ATF6, which contributes to the activation of ATF6 (Fig. 7; Nadanaka *et al.* 2007; Oka *et al.*, 2019).

It is therefore possible that ATF6 acts as a sensor for the ER redox status. One counterargument against this idea comes from the fact that ATF6 is reduced not only by DTT-induced stress but also in response to other ER-stressing stimuli that do not seem to directly alter the ER redox status (Nadanaka *et al.*, 2006; Nadanaka *et al.*, 2007). The ER may have a sophisticated machinery that stimulates the ATF6 reduction in response to ER stress even without global variation in the ER redox status. Indeed, according to Oka *et al.* (2019), an ER‐resident oxidoreductase, ERp18, transiently associates with ATF6α upon ER stress, assisting the activation of ATF6. The protein disulfide isomerase A5 is also likely to contribute to the reduction and activation of ATF6 (Higa *et al.*, 2014). Meanwhile, it should also be noted that the ability of the ER to form protein disulfide bonds can be impaired commonly by ER-stressing stimuli including those that do not seem to directly lead to the reduction of ER proteins (Merksamer *et al.*, 2008).

How does the reduction of ATF6 lead to its activation as a soluble and active transcription factor? Nadanaka *et al.* (2007) proposed that the ATF6 reduction contributes to (but is not sufficient for) its transport to the Golgi apparatus. An observation contradicting this scenario is that the ER-to-Golgi transport of ATF6 was promoted by the ERp18 knockout mutation (Oka *et al.*, 2019). Meanwhile, it is also likely that the ATF6 reduction is required for its correct cleavage in the Golgi apparatus (Nadanaka *et al.*, 2007; Oka *et al.*, 2019). Fig. 7 shows a current model for the activation of ATF6 in response to ER stress. In agreement with this model, Sundaram *et al.* (2018) reported that ER stress deceases the oligomerization size (homo- or hetero-oligomers) of ATF6.

Similarly to the Ire1 orthologues and PERK, ATF6 may sense LBS through a molecular mechanism different from that for the sensing of disturbance in ER protein folding. Tam *et al.* (2018) reported the activation of ATF6 by cellular treatment with dihydrosphingosine and dihydroceramide. In this case, the stress-sensing module is likely to lie not on the luminal domain but on the transmembrane domain of ATF6 (Tam *et al.*, 2018).

### Conclusion and future perspective

As described here, yeast Ire1 is controlled in multiple fashions, which include negative regulation by BiP and the direct capturing of unfolded proteins. Although seemingly understood better than that of IRE1α, the regulation mechanisms of yeast Ire1 still present unresolved questions. For instance, it remains unclear how yeast Ire1 escapes from repression by BiP upon LBS. It is also possible that yeast Ire1 undergoes an as-yet undisclosed regulatory event. For instance, according to Wiseman *et al.* (2010), the cytosolic domain of yeast Ire1 has a putative ligand-binding pocket that can control its RNase activity.

Regulatory processes for IRE1α seem to be more complicated than those for yeast Ire1, and remains controversial points. It should be determined in future to what extent the direct interaction between IRE1α and unfolded proteins contributes to the regulation of IRE1α in response to ER stress. Besides, unlike yeast Ire1, IRE1α proximately evokes multiple events, including the RIDD and the XBP1-mRNA splicing. Moreover, not only the RNase activity but also the kinase activity of IRE1α is likely to directly act to induce downstream events in mammalian cells (Urano *et al.*, 2000; Nishitoh *et al.*, 2002; Ali *et al.*, 2011; Hetz and Papa, 2018).

Intriguingly, the activities of IRE1α to promote RIDD and *XBP1*-mRNA splicing are differently regulated (Han *et al.*, 2009). One scenario is that dimerized IRE1α molecules perform only *XBP1*-mRNA splicing, whereas further oligomerization of IRE1α is required for its RIDD activity (Han *et al.*, 2009; Ghosh *et al.*, 2014; Bashir *et al.*, 2020). Whereas *XBP1*-mRNA splicing by IRE1α mainly acts cytoprotectively, RIDD can trigger apoptosis, for instance, through the digestion of microRNAs that repress translation of the mRNA encoding Caspase 2, an apoptosis initiator (Upton *et al.*, 2012; Maurel *et al.*, 2014). Although other scenarios are also possible (Iurlaro and Muñoz-Pinedo, 2016; Bashir *et al.*, 2020), these insights explain a mechanism for apoptosis induction by strong and chronic ER stress. That is, although mild ER stress leads only to the dimerization of IRE1α, which eventually rescues the cells, strong and chronic ER stress causes severe accumulation of unfolded proteins in the ER, which triggers the proapoptotic RIDD through high-order oligomerization of IRE1α. Moreover, the regulatory mechanism of IRE1α’s ability to phosphorylate other proteins is another interesting question, since, as aforementioned, IRE1α is likely to act as a kinase but not as an RNase for activation of JNKs, which also leads to apoptosis (Sano and Reed, 2013).

Another implication of the insights described so far is that, although collectively called ER-stress sensors, IRE1α, PERK, and ATF6 sense stressing stimuli and are regulated in different manners. We think that this issue can be a molecular basis for activating these proteins on different timelines in ER-stressed cells (Yoshida *et al.*, 2003). Moreover, these ER-stress sensors carry out different physiological roles in multicellular organisms (Mitra and Ryoo, 2019), probably and partly because they are regulated differently.

## Abbreviations

AH: amphipathic helix
ATF: activating transcription factor
ATF6(C): C-terminal fragment of ATF6
ATF6(N): N-terminal fragment of ATF6
CHOP: C/EBP homologous protein
cLD: core luminal domain
COPII: coat protein complex II
CSSR: core stress-sensing region
DTT: dithiothreitol
eIF2α: α subunit the eukaryotic initiation factor 2
ER: endoplasmic reticulum
*HAC1*i: induced *HAC1*
*HAC1*u: uninduced *HAC1*
HSP: heat shock protein
JIDR: juxtamembrane intrinsically disordered region
JNK: c-Jun N-terminal kinase
LBS: lipid bilayer stress
NBD: nucleotide-binding domain
NBP: nucleotide-binding pocket
NUCR: N-terminal unconserved region
PERK: PKR-like endoplasmic reticulum kinase
PC: phosphatidyl choline
RNase: (endo)ribonuclease
RIDD: regulated Ire1-dependent decay
SBD: substrate-binding domain
TMH: transmembrane helix
UPR: unfolded protein response
VLCFA: very-long-chain fatty acid
XBP1: X-box binding protein 1
XBP1s: spliced XBP1
XBP1u: unspliced XBP1
